# Parenting styles and obsessive-compulsive symptoms in college students: the mediating role of perfectionism

**DOI:** 10.3389/fpsyt.2023.1126689

**Published:** 2023-07-07

**Authors:** Po Hu, Pengwei Liang, Xiaoyan Liu, Yuting Ouyang, Jianping Wang

**Affiliations:** ^1^School of Educational Sciences, Xuzhou University of Technology, Xuzhou, China; ^2^Beijing Key Laboratory of Applied Experimental Psychology, Faculty of Psychology, National Demonstration Center for Experimental Psychology Education (Beijing Normal University), Beijing Normal University, Beijing, China

**Keywords:** obsessive-compulsive symptoms, perfectionism, parenting styles, college students, mediation analysis

## Abstract

**Introduction:**

Obsessive-compulsive symptoms is a common psychological phenomenon among early adulthood college students, which are closely related to their parents’ parenting styles. Theoretical and some empirical studies have suggested the mediating role of perfectionism in this process, but this has not been confirmed, and the binary perspective of positive-negative perfectionism has not been addressed in this issue.

**Methods:**

This study aimed to investigate the mediating role of perfectionism in the relationship between parenting styles and obsessive-compulsive symptoms among college students. A total of 661 college students participated in this study.

**Results:**

Negative perfectionism mediated the relationship between negative parenting style and obsessive-compulsive symptoms among college students. Negative parenting style predicted obsessive-compulsive symptoms through its positive predictive effect on negative perfectionism. However, the mediating effect of positive perfectionism was not significant in this process.

**Discussion:**

This study provides further evidence of the mechanisms underlying the occurrence of obsessive-compulsive symptoms, and offers new ideas for interventions for OCD.

## 1. Introduction

### 1.1. The direct effect of parenting styles on obsessive-compulsive symptoms

Obsessive-compulsive-related disorders are distinguished from other disorders in the Diagnostic and Statistical Manual of Mental Disorders, Fifth Edition (DSM-5), which lists obsessive-compulsive thoughts and compulsive behaviors as two parallel components that together constitute obsessive-compulsive disorder. Muris found that obsessive-compulsive symptoms are prevalent in the general population, and that these symptoms can be recurrent and persistent (1). In fact, over 80% of the general population report compulsive behavior, such as repeatedly checking that car and house doors are locked, and to see if they have forgotten something; such behaviors are not fundamentally different from the compulsive behavioral habits of clinical patients (2). This suggests that the main difference between normal people and those with OCD is one of degree rather than kind. As such, certain compulsive symptoms can occur in normal people.

A large sample of epidemiological surveys conducted in Greece showed that 1.69% of participants met the diagnostic criteria for OCD and 2.79% exhibited subclinical symptoms of OCD (3). In China, measurements using the SCL-90 show that the scores of obsessive-compulsive symptoms in the population measured in 2015 were significantly higher than the norm obtained in 1986, especially in women, with the proportion of moderate and above reaching 20.5% (4). A cross-national survey shows that the average age of onset of OCD is 17.9 years old, generally in late adolescence, just as Chinese students are entering college (5). Thus, OCD onsets early, at an age that coincides with university education. Accordingly, OCD is one of the most common psychological disorders among university students, and most OCD patients on university campuses are initial-onset cases. The prevalence and persistence of obsessive-compulsive symptoms associated with the physical and mental health of university students (6).

Jia (7) used the Symptom Checklist 90 (SCL-90) to investigate the mental health of university freshmen. A mental health survey using the SCL-90 shows that 29.5% of medical college students in China experienced obsessive-compulsive symptoms, and obsessive-compulsive symptoms were the highest percentage of positive symptoms detected (8). A study in Spain showed that female university students scored significantly higher on the obsessive-compulsive subscale of the SCL-90 than the average for women of all ages (9). A newer study showed that 30% of the college student population scored highly for obsessive-compulsive symptoms (10). The above studies indicate that obsessive-compulsive symptoms are prevalent on college campuses and are strongly associated with mental health problems and impairment of social functioning among college students (11–13).

Given that obsessive-compulsive symptoms tend to onset in early adulthood, researchers have focused on one of the most important external factors associated with the development of individuals during adolescence, i.e., parenting styles (14–17). define parenting style as a constellation of parental attitudes, emotions, and behaviors toward raising children that accumulate over time in parents during the process of childrearing. This construct is relatively stable and does not tend to change from situation to situation. The Swedish clinical psychologist Perris et al. (18) developed the EMBU (Egna Minnen av. Barndons Uppforstran) a parenting style questionnaire. The standard version of the EMBU is divided into two sections (one each for fathers and mothers) and has four core dimensions: Rejection, Emotional Warmth, Overprotection, and Favoring Subject. The EMBU scale has been validated in various cultural groups. In the revised Chinese version of the scale, parenting styles were aggregated into six dimensions for fathers and five dimensions for mothers, totaling 11 dimensions (19). Two dimensions were identified as positive parenting styles, while the remaining nine dimensions reflected negative parenting styles.

Researchers found a significant positive correlation between obsessive-compulsive symptoms and poor parenting (20, 21). Adolescents who experience more verbal and physical hostility from their parents (a characteristic of authoritarian parenting styles) have higher levels of anxiety as adults, exhibit more severe obsessive-compulsive symptoms, and are at higher risk for developing obsessive-compulsive disorder. (14, 22, 23). There are also review articles pointing to a strong correlation between poor parenting practices and various types of psychological and psychiatric problems in children (e.g., obsessive-compulsive symptoms, depression, hostility, poor interpersonal relationships, paranoia, psychoticism, etc.) (24). Severe stress and family dysfunction are commonly seen in families of adolescents and college students who exhibit obsessive-compulsive symptoms (23, 25). Based on the above, it is evident that parenting style can greatly influence the severity of obsessive-compulsive symptoms in children.

### 1.2. The mediating effect of perfectionism

Perfectionism has been associated with obsessive-compulsive symptoms, both theoretically and in actual observations, and is thus considered a risk factor for obsessive-compulsive symptoms (26, 27). The Integrated Cognitive Model (ICM) follows Beck’s cognitive theory, which suggests that personal life events and experiences influence a person’s beliefs and greatly impact their perception and evaluation of reality. The psychological mechanism of obsessive-compulsive symptoms is closely related to the dysfunction of cognitive functions, and the symptoms are rooted in perfectionist beliefs (28, 29) stated that perfectionism manifests itself in the short term as a set of negative cognitive traits and behavioral responses, and in the long term as negative personality traits, as evidenced by an excessive reliance on the achievement of extremely high standards in at least one domain for self-worth, accompanied by an excessive focus on mistakes and harsh self-criticism. The above cognitive process is very similar to obsessive-compulsive symptoms, such as the demand of perfectionists to perform perfectly in learning, life, and even work, which is essentially consistent with the zero-tolerance attitude toward imperfection of individuals with obsessive-compulsive symptoms.

As research on perfectionism continues to accumulate, it is now generally agreed that perfectionism, as a psychological phenomenon, is not a unidimensional structure. Terry-Short et al. (30) suggested that perfectionists can be divided into two groups, and developed a scale to distinguish positive and negative perfectionism. In the former group, perfectionist behavior is carried out in pursuit of positive goals, whereas in the latter, perfectionist behavior is performed to avoid negative consequences or responsibility. The most representative conceptualization is the six-dimensional model of Frost et al. (31). Based on this, Frost developed the Frost Multidimensional Perfectionism Scale (FMPS) to measure typical cognitive, behavioral, and emotional expressions of perfectionists. Five dimensions belong to negative perfectionism, and the remaining one is positive perfectionism. The two-dimensional perspective of perfectionism has also been validated in empirical research, further enriching the relationship between perfectionism and maladaptive psychological outcomes. A series of studies have shown that positive perfectionism is more likely to be associated with positive outcomes, while negative perfectionism has the opposite effect [i.e., (32, 33)].

Numerous studies have shown that parenting styles play a crucial role in shaping children’s personalities, especially in the formation of perfectionist personalities, (31, 34, 35). Perfectionism is largely learned and may be influenced by parenting styles and the familial environment during childhood (36–39). Barret et al. (40) proposed an anxious parenting model to explain the development of perfectionism in children. According to their model, anxious parents are overly sensitive to, and overly concerned about, their children’s mistakes. Thus, the children will try to avoid mistakes to meet their parents’ demands or expectations. The model posits a vicious circle, in which parents’ excessive anxiety inevitably leads to over-interference or over-protection of their children, under the guise of it being for their own good, to prevent mistakes. In the long run, however, this leads to the development of perfectionist personality traits. If parents teach their children that perfection and success is the key to love and affection, children who do not meet expectations are more likely to develop maladaptive perfectionism by internalizing the associated negative self-evaluation (41). Results of a survey conducted in a sample group of undergraduates suggest that early parenting practices may be a precursor to the development of perfectionism (42). According to the transdiagnostic perspective, some pathogenic mechanisms in psychological disorders may share common cognitive processes, and perfectionism is likely to have transdiagnostic features, with self-reported perfectionism scores of OCD patients differing from those of healthy control (43). Perfectionism explains obsessive-compulsive symptoms from a different perspective (44). Individuals with high perfectionism set excessively high and unrealistic standards for themselves, and constantly strive to achieve them. This is very similar to obsessive-compulsive symptoms (45). Although there is evidence from both theoretical and empirical perspectives that there is a positive relationship between perfectionism, parenting styles, and obsessive-compulsive symptoms, we still believe it is necessary to further explore the relationship between perfectionism and obsessive-compulsive symptoms from the perspective of positive–negative perfectionism. This can help us better understand the cognitive processes of individuals with obsessive-compulsive symptoms. Furthermore, when we understand this issue from the perspective of binary perfectionism, we can also recognize that perfectionism is adaptive to some extent.

### 1.3. Overview of study

In summary, obsessive-compulsive symptoms occur in a high proportion of Chinese college students and are associated with their psychological health. Unfortunately, the timeliness of treatment is often suboptimal. In both Western and Eastern countries, the time lag between the onset of noticeable obsessive-compulsive symptoms and professional treatment at a facility for OCD patients is between 5 and 7 years (46, 47). This reflects the fact that OCD patients’ symptoms are not fundamentally different from those of normal healthy groups, and also reflects the lack of awareness and attention given to obsessive-compulsive symptoms by society. In addition, previous studies have focused on the pathology and treatment of OCD, with little research being done on subclinical samples and college students with similar symptoms. Therefore, this study will focus on the obsessive symptoms of college students in non-clinical samples.

To date, studies on college students have mainly explored the relationship between only two of the following factors: obsessive-compulsive symptoms, perfectionism, and parenting styles. However, few studies have been conducted in non-clinical samples in China to examine the mechanisms of parenting styles on the development of obsessive-compulsive symptoms in college students. Therefore, this study explored the predictive role of parenting style on OCD symptoms in college students and the mediating role of perfectionism on the pathway. The study proposed the following hypotheses:

*Hypothesis 1*: Poor parenting style positively predicts obsessive-compulsive symptom scores.

*H1a*: Poor paternal parenting positively predicts obsessive-compulsive symptoms.

*H1b*: Poor maternal parenting positively predicts obsessive-compulsive symptoms.

*Hypothesis 2*: Perfectionism mediates the role of poor parenting style on obsessive-compulsive symptoms.

*H2a*: Perfectionism mediates the role of poor fathering styles on obsessive-compulsive symptoms.

*H2b*: Perfectionism mediates the role of poor maternal parenting on obsessive-compulsive symptoms.

## 2. Methods

### 2.1. Participants and procedures

The participants in this study were 661 university students from two Chinese universities. The mean age of the participants was 18.6 ± 0.92 years. 404 (61.1%) were male and 257 were female (38.9%). The test was administered by a researcher during a mental health education class. The participants were first provided with instructions regarding the questionnaire answer options and informed consent. The participants then filled out the questionnaire, which took about 20 min.

### 2.2. Measures

#### 2.2.1. Obsessive-compulsive symptoms

The Obsessive-Compulsive Scale—Revised (OCI-R) was developed by Foa et al. (2002) (48). It is a shortened version of the Obsessive-Compulsive Scale (OCI) and has a total of 18 items. Each item is divided into frequency of occurrence and distress components, and is scored on a five-point scale (range: 0–4). The frequency of occurrence scores range from 0 (*Never*) to 4 (*Almost always*). Distress scores range from 0 (*Not at all distressing*) to 4 (*Extremely distressing*). The Chinese version of the OCI-R is divided into six dimensions: namely Washing, Checking, Ordering, Obsessing, Hoarding and Mental Neutralizing. The alpha coefficients for the six dimensions are 0.50, 0.58, 0.63, 0.56, 0.57, and 0.59 respectively, and the internal consistency coefficient for the entire scale is 0.90.

#### 2.2.2. Parenting style

The Chinese version of the Parenting Style Scale (Egna Minnen av. Barndons Uppforstran, EMBU) was used in this study. The scale was revised by Yue et al. (19) based on the Parenting Style Questionnaire developed in Sweden by Perris et al. (18), and consists of 66 items (11 dimensions).Father’s parenting style has six dimensions: emotional warmth and understanding, punishment and severity, overprotection, favoritism, rejection and denial, and excessive protection. Mother’s parenting style has five dimensions: emotional warmth and understanding, excessive protection and interference, rejection and denial, punishment and severity, and favoritism. The dimension of emotional warmth and understanding is positive parenting style, while the rest are negative parenting styles. The Chinese version of the EMBU is scored on a four-point scale ranging from 1 (*never*) to 4 (*always*). The alpha coefficients for the dimensions of the father’s parenting style in this study were 0.86, 0.86, 0.67, 0.75, 0.64, and 0.65 respectively; the alpha coefficients for the dimensions of the mother’s parenting style were 0.86, 0.73, 0.75, 0.85, and 0.74.

#### 2.2.3. Perfectionism

The original English version of the CFMPS (The Chinese Frost Multidimensional Perfectionism Scale) was developed by Frost et al. (31) to measure the typical cognitive, behavioral, and emotional characteristics of perfectionists, and is one of the most commonly used instruments for measuring perfectionism.

The CFMPS contains 27 questions (five dimensions). The five dimensions are as follows: Concern Over Mistakes (CM), Parental Expectations (PE), Personal Standards (PS) and Doubt About Actions (DA), and Organized (OR). The scoring scale ranges from 1 (*non-conformity*) to 5 (*conformity*). Dimension scores are summed to obtain the total score. According to Fei and Zhou (49) and Parker (1997), perfectionism can be classified as positive or negative, where CM, PE, PS, and DA reflect negative perfectionism and OR positive perfectionism. In this study, the alpha coefficients for each dimension were: 0.81, 0.74, 0.75, 0.64, 0.82.

### 2.3. Data analysis

The data were analyzed using SPSS (ver. 18.0; SPSS Inc., Chicago, IL, United States) and Mplus 7.0. Missing values (<10%) were accounted for using expectation maximization. For correlation and regression analyses, structural equation modeling was used to test for possible indirect effects of negative perfectionism on the relationship between obsessive-compulsive symptoms and parenting style. Mediating effects were identified using the bias-corrected percentile bootstrap method (50). If no paths included 0 in their 95% confidence interval, a mediating effect was considered present, and vice versa (51, 52).

## 3. Results

Descriptive statistics and correlation analysis results are shown in Table 1. As there was no significant correlation found between positive perfectionism (Organized), obsessive-compulsive symptoms, positive parenting styles, parental favoritism, and negative perfectionism, these variables were not included in subsequent analyses.

**Table 1:** Descriptive statistics and correlation analysis results.

	*M*	SD	1	2	3	4	5	6	7	8	9	10	11	12	13	14	15	16
Obsessive-compulsive symptoms	10.79	9.06	–															
**Paternal parenting style**																		
Emotional warmth and understanding	52.08	8.76	−0.138^**^	–														
Punishment severity	15.5	4.58	0.306^**^	−0.252^**^	–													
Excessive interference	19.99	3.77	0.144^**^	0.153^**^	0.491^**^	–												
Rejection and denial	8.97	2.6	0.256^**^	−0.255^**^	0.670^**^	0.476^**^	–											
Overprotective	10.6	2.52	0.212^**^	0.120^**^	0.421^**^	0.512^**^	0.409^**^	–										
**Maternal parenting style**																		
Emotional warmth and understanding	53.02	7.97	−0.119^**^	0.805^**^	−0.275^**^	0.076	−0.287^**^	0.036	–									
Overprotection and excessive interference	34.86	6.13	0.236^**^	0.036	0.417^**^	0.606^**^	0.409^**^	0.564^**^	0.051	–								
Rejection and denial	12.5	3.58	0.290^**^	−0.273^**^	0.605^**^	0.321^**^	0.653^**^	0.305^**^	−0.330^**^	0.532^**^	–							
Punishment severity	12.08	3.8	0.282^**^	−0.239^**^	0.761^**^	0.295^**^	0.513^**^	0.306^**^	−0.307^**^	0.460^**^	0.713^**^	–						
Concern over mistakes	12.56	5.08	0.365^**^	−0.084^*^	0.220^**^	0.155^**^	0.226^**^	0.227^**^	−0.083^*^	0.265^**^	0.230^**^	0.211^**^	–					
Parental expectations	14.68	4.77	0.177^**^	0.009	0.278^**^	0.302^**^	0.215^**^	0.316^**^	−0.02	0.373^**^	0.250^**^	0.235^**^	0.299^**^	–				
Personal standards	16.95	5.12	0.242^**^	0.069	0.213^**^	0.142^**^	0.175^**^	0.274^**^	0.061	0.298^**^	0.178^**^	0.195^**^	0.518^**^	0.362^**^	–			
Doubt about actions	12.51	3.55	0.348^**^	−0.06	0.174^**^	0.126^**^	0.153^**^	0.226^**^	−0.041	0.243^**^	0.179^**^	0.163^**^	0.418^**^	0.248^**^	0.350^**^	–		
Positive perfectionism (organized)	22.65	4.93	0.03	0.182^**^	−0.124^**^	−0.048	−0.113^**^	0.002	0.159^**^	−0.013	−0.097^*^	−0.096^*^	−0.007	0.07	0.249^**^	0.109^**^	–	
Negative perfectionism	56.7	13.52	0.382^**^	−0.018	0.307^**^	0.252^**^	0.267^**^	0.360^**^	−0.026	0.408^**^	0.289^**^	0.279^**^	0.788^**^	0.667^**^	0.793^**^	0.640^**^	0.145^**^	–

**Figure 1:**
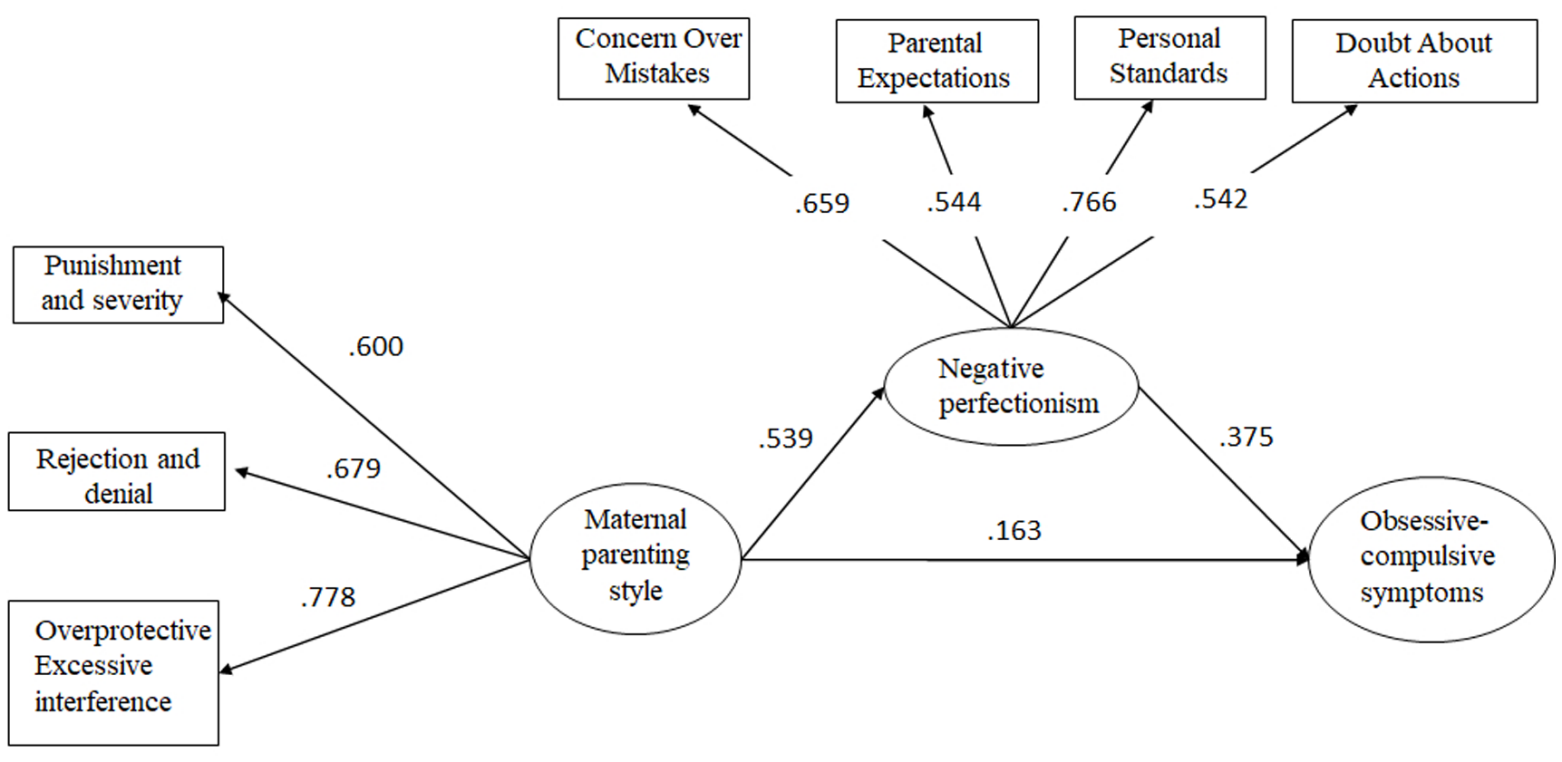
The mediating role of maternal parenting style in the relationship between negative perfectionism and obsessive-compulsive symptoms.

The indirect effect of maternal parenting style on the relationship between negative perfectionism and obsessive-compulsive symptoms was tested first. The results were as follows: *χ*^2^/df = 5, CFI = 0.948, TLI = 0.910, RMSEA = 0.078 (90% confidence interval: 0.063–0.093), and SRMR = 0.047; all fit indices met the significance criterion, indicating good model fit. Further examination of the model’s parameter estimates revealed that all path coefficients were significant (*ps* < 0.05; Figure 1).

**Figure 2:**
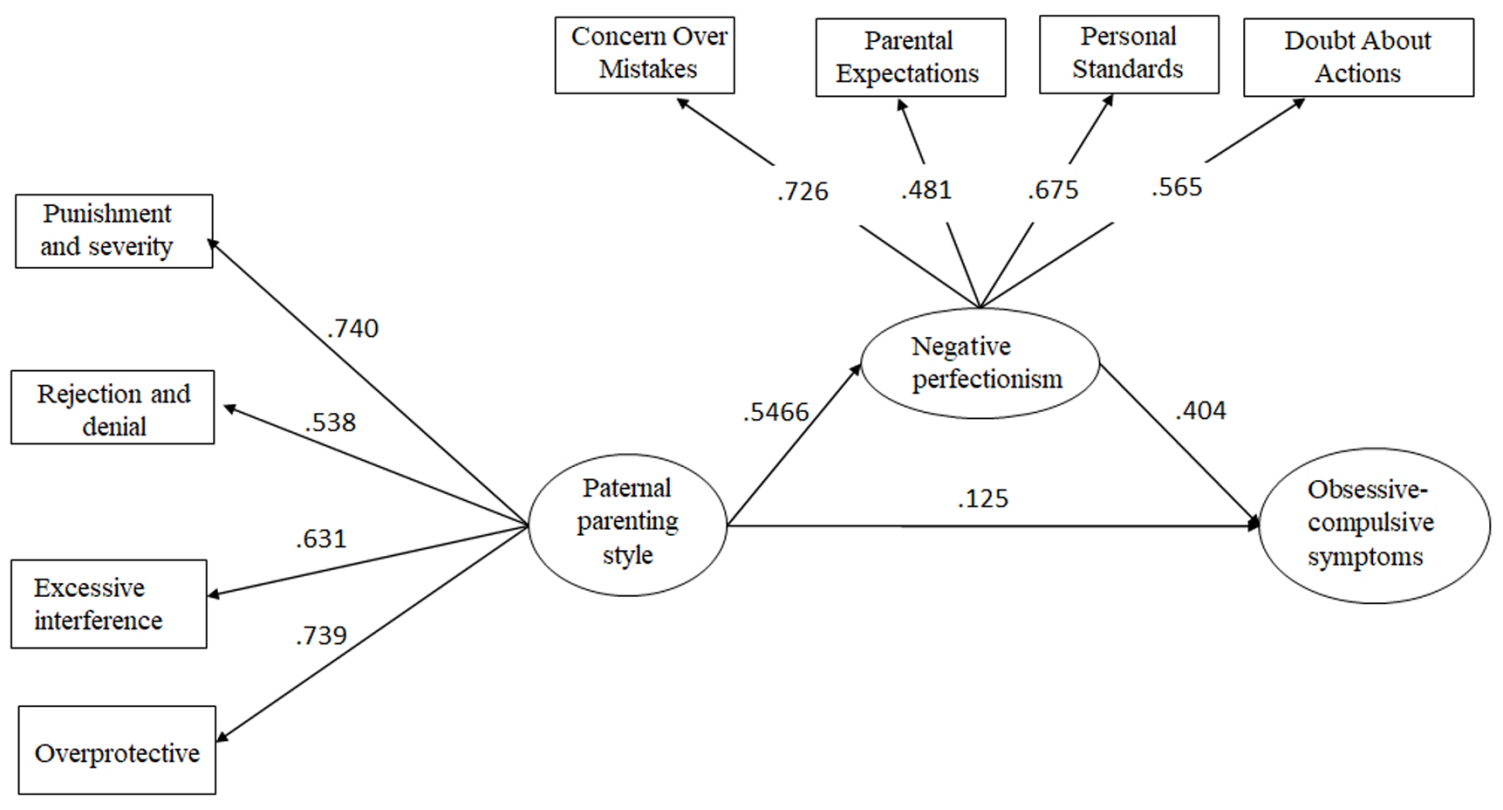
The mediating role of paternal parenting style in the relationship between negative perfectionism and obsessive-compulsive symptoms.

Next, the indirect effect of paternal parenting style on the relationship between negative perfectionism and obsessive-compulsive symptoms was examined; the results were as follows: *χ*^2^/df = 4.63, CFI = 0.960, TLI = 0.930, RMSEA = 0.074 (90% confidence interval: 0.058–0.092), and SRMR = 0.04. All of the fit indices met the significance criterion, indicating a good model fit. Further examination of the model parameter estimates revealed that all path coefficients were significant (*ps* < 0.05; Figure 2).

The Bootstrap procedure was used to test the significance of the mediating effect of negative perfectionism on the relationship between obsessive-compulsive symptoms and parenting style (52) (Table 2).

**Table 2:** Bootstrap analysis of the mediating effect of parenting style.

Pathways	Standardized Indirect effects estimates	95% confidence interval	
		Lower bound	Upper bound
Maternal parenting style—negative perfectionism—obsessive-compulsive symptoms	0.188	0.122	0.255
Paternal parenting style—Negative perfectionism—Obsessive-compulsive symptoms	0.193	0.121	0.265

## 4. Discussion

This study examined the relationship between parenting style and obsessive-compulsive symptoms in university students. Understanding and positive warmth were negatively associated with obsessive-compulsive symptoms, indicating that the more positive the parenting style, the less mental health problems during development. In contrast, negative parenting styles were significantly positively associated with obsessive-compulsive symptoms, meaning that the more negative parenting, the more severe the obsessive-compulsive symptoms appeared in the child. This is consistent with a review of previous literature showing that poor parenting styles are highly correlated with various types of psychological and psychiatric problems in children (e.g., obsessive-compulsive symptoms, depression, hostility, poor interpersonal relationships, paranoia, psychoticism, etc.) (14, 23–25).

In addition to the direct effect of parenting styles on obsessive-compulsive symptoms, this study is more concerned with the mediating effect of perfectionism in this relationship, as well as whether there are differences in the role of positive and negative perfectionism. As we hypothesized, positive and negative perfectionism play different roles. For negative perfectionism, this study found that it mediates the relationship between negative parenting styles and obsessive-compulsive symptoms, while positive perfectionism is not significantly related to obsessive-compulsive symptoms. That is, the more negative the parenting style, the more severe the negative perfectionism of college students, and thus the more severe their obsessive-compulsive symptoms. Numerous empirical studies have confirmed that the formation and shaping of perfectionist personalities in children are strongly influenced by parenting styles (30, 35, 41, 42, 48). For example, the more harshly parents criticize their children, the higher the standards set, and the greater the lack of emotional warmth and understanding, the stronger the child’s tendency toward negative perfectionism will be (35). Negative parenting styles are likely to contribute to the development of negative perfectionism in university students. Inappropriate parenting and communication styles may reflect a low level of tolerance for children’s mistakes, which may lead the children to believe that they have to avoid making mistakes; thus, negative perfectionist traits develop. In terms of the relationship between perfectionism and obsessive-compulsive symptoms, the present study found a significant positive association between negative perfectionism and obsessive-compulsive symptoms, similar to the findings of many studies conducted outside of China (27, 43–45). This suggests that, for university students, an individual’s pursuit of perfection may be accompanied by an increase in intrusive thoughts associated with perfectionism (e.g., “I must do everything perfectly and impeccably”), thus leading to perfectionist behaviors and obsessive-compulsive symptoms. In addition, another interesting finding of this study is that the relationship between positive perfectionism and obsessive-compulsive symptoms is not significant. In other words, not all pursuits of organization, order, and perfect task completion are related to maladaptive obsessive-compulsive symptoms in college students. This dichotomous relationship between perfectionism and psychological adaptation is similar to the conclusions of previous research (33). This also suggests that the desire for things to be done perfectly is not necessarily maladaptive to some extent. Finally, it is worth noting that in the two mediation models proposed in this study, the model of maternal parenting behavior has a larger effect size, which is consistent with the cultural background of the participants in this study. In Chinese culture, it is the norm for men to be the breadwinners and women to be the caregivers at home, so mothers are more involved in their children’s education (53). In addition, in contemporary Chinese culture, the term “Tiger Mom, Cat Dad” has emerged, suggesting that mothers play a more strict and demanding role in family education (54), which makes the strong relationship between maternal parenting style and child OCD symptoms more plausible. As a result, the relationship between a mother’s negative parenting style and a child’s perfectionism and obsessive-compulsive symptoms is more closely related. In clinical practice, psychological work with university students mostly involves only the associated with individual, and not the parents. However, the findings of this study suggest that interventions involving the parents of college students with obsessive-compulsive symptoms, aimed at improving their parenting style, could reduce obsessive-compulsive symptoms. In addition, interventions to reduce negative perfectionism and obsessive-compulsive symptoms appear feasible. In this study, negative perfectionism comprised four dimensions (CM, PE, PS, and DA). A psychological intervention aimed at addressing negative perfectionism according to the above four dimensions may be effective in reducing obsessive-compulsive symptoms and subjective distress.

Finally, this study had certain shortcomings that should be addressed in future research. First, parenting styles were examined using the EMBU, and the results may have been influenced by the cognitive processes of the participants. Research methods such as observation and coding could be used in future studies to increase the validity of the findings. Second, although the OCI-R, a tool specifically used for assessing OCD, was employed in this study, no clinical sample was included and the applicability of the results to clinical populations warrants further research. Finally, due to the cross-sectional design of this study, causality could not be inferred. Future studies should adopt a longitudinal design to examine the long-term effects of parenting styles on perfectionism.

## Data availability statement

The raw data supporting the conclusions of this article will be made available by the authors, without undue reservation.

## Ethics statement

The studies involving human participants were reviewed and approved by Ethics Committee of Beijing Normal University. The patients/participants provided their written informed consent to participate in this study.

## Author contributions

PH, PL, and JW: study design. PH: data collection. PL and XL: data analysis. PH, PL, XL, and YO: manuscript writing. JW: study supervision. All authors contributed to the article and approved the submitted version.

## Funding

This study was supported by the Social Science Research Project Fund of Jiangsu Province (Project No. 2019SJB922).

## Conflict of interest

The authors declare that the research was conducted in the absence of any commercial or financial relationships that could be construed as a potential conflict of interest.

## Publisher’s note

All claims expressed in this article are solely those of the authors and do not necessarily represent those of their affiliated organizations, or those of the publisher, the editors and the reviewers. Any product that may be evaluated in this article, or claim that may be made by its manufacturer, is not guaranteed or endorsed by the publisher.
